# Nationwide seroprevalence of paratuberculosis in indigenous Turkish goat breeds reveals extensive herd-level circulation and breed-associated risk

**DOI:** 10.1186/s13620-026-00349-z

**Published:** 2026-06-16

**Authors:** Yalçın Yaman, Ahmet Eser, Devran Coşkun, Ramazan Aymaz, Yiğit Emir Kişi, Murat Keleş, Serdar Yağcı, Özgül Gülaydın, Serkan Süleyman Şengül, Kıvanç İrak, Memiş Bolacalı

**Affiliations:** 1 Faculty of Veterinary Medicine, Siirt University, Siirt, TR-56100 Türkiye; 2 Department of Breeding Techniques, Sheep Breeding Research Institute, Bandırma, Balıkesir, TR-10200 Türkiye; 3 Department of Livestock and Aquatic Products, General Directorate of Agricultural Research and Policies, Ankara, TR-06000 Türkiye; 4 Department of Biostatistics and Medical Informatics, Faculty of Medicine, Kırşehir Ahi Evran University, Kırşehir, TR-40100 Türkiye

**Keywords:** Paratuberculosis, MAP, Johne’s disease, Seroprevalence, Turkiye, Indigenous goat breeds, ELISA, Modified Poisson regression, Prevalence ratio, Population attributable fraction

## Abstract

Paratuberculosis (Johne’s disease), caused by *Mycobacterium avium* subsp. *paratuberculosis* (MAP), imposes substantial veterinary burdens globally, with potential zoonotic implications, yet systematic surveillance in Turkish goat populations has been poorly documented. This study presents the first national, multi-breed cross-sectional serosurvey of MAP infection across seven indigenous Turkish goat breeds (*n* = 3,372; 36 herds; 11 provinces), employing a commercial indirect ELISA and modified Poisson regression with robust variance estimation to generate prevalence ratios (PRs) directly interpretable as relative risks. Overall apparent seroprevalence reached 14.07% (95% CI: 12.83–15.31%), with herd-level positivity of 93.5%—establishing endemic dissemination across geographically and climatically diverse production systems. Breed-level seroprevalence spanned a 2.66-fold gradient (8.45%–22.47%). Four breeds showed significantly elevated adjusted PRs: Colored Angora (PR = 2.702), Turkish Saanen (PR = 2.571), Maltese (PR = 2.027), and Honamlı (PR = 1.469); three retained significance under cluster-robust estimation. The intraclass correlation coefficient (ICC = 0.031) indicated low within-herd clustering, suggesting that individual-level factors may contribute more to seroconversion outcome than shared herd environment in this dataset. Population attributable fraction analysis identified Turkish Saanen (19.70%) and Maltese (13.77%) as the dominant drivers of national disease burden. The seroprevalence contrast between White Angora (0%) and Colored Angora (22.47%) warrants controlled investigation of differential exposure, management, and potentially breed-specific factors. These findings provide an evidence base for establishing a national MAP control programme for small ruminants in Türkiye.

**Supplementary Information:**

The online version contains supplementary material available at 10.1186/s13620-026-00349-z.

## Introduction

Paratuberculosis, commonly referred to as Johne’s disease (JD), is a chronic granulomatous enteritis of domestic and wild ruminants caused by *Mycobacterium avium* subsp. *paratuberculosis* (MAP) [1], with the etiologic role of MAP established across cattle, sheep, goats, deer, and a broader wildlife context [1–3]. The disease is globally distributed and imposes significant economic losses through reduced productivity, premature culling, and secondary health complications [1, 4, 5]. MAP infection is characterised by a prolonged subclinical phase [6] during which persistent infection endures despite host immune responses [1, 7, 8] and subclinical animals shed MAP faecally, sustaining environmental contamination [7, 9]; MAP can additionally be shed via colostrum and milk, raising zoonotic concerns [4, 9, 10]. Spore-like MAP structures resist pasteurisation and environmental degradation for months, and viable MAP has been detected in infant formula [11–13]; insect vectors constitute a further transmission route [14]. MAP’s host range extends into non-ruminant wildlife — foxes, stoats, lagomorphs, wild boar — documented as reservoirs in shared habitats [7, 15–20], with cross-species transmission dynamics reported across Europe and elsewhere [7, 16–20].

The potential association between MAP and Crohn’s disease (CD) in humans, while epidemiologically and mechanistically discussed, remains causally unresolved [1, 2, 10, 21, 22]; nonetheless, MAP’s zoonotic potential via dairy products and environmental routes has prompted ongoing food-safety and public health scrutiny [4, 10, 23, 24]. Economically, JD imposes losses estimated between hundreds of millions and over one billion dollars annually in the United States [25, 26], arising from reduced milk yield, premature culling, and decreased slaughter value [27, 28]; infected animals can remain asymptomatic for years while productivity declines [29, 30], and these hidden losses contribute to tens of billions in annual dairy disease costs globally [31]. European losses similarly reach hundreds of millions annually [25, 31, 32]. Diagnostic test insensitivity during early infection has historically undermined control programme uptake [33], underscoring the need for integrated approaches combining surveillance, vaccination, and genetically informed breeding [4, 5, 31, 34–36].

Paratuberculosis is endemic globally: herd-level cattle prevalence approximates 20% in Europe [37], with persistent infection in Germany and Poland [38, 39]; 15–50% in Asia [40–42]; 8.8% in Uganda, with higher estimates elsewhere in Africa [43, 44]; up to 55% in North American dairy herds [4, 45]; and approximately 16% in South America [37, 46]. In small ruminants, sheep seroprevalence reaches approximately 4% in parts of Europe [37, 47, 48], up to 15% in India [40], with lower figures in the Americas [49, 50]. Goat prevalence is notably higher: 21–54% in Europe, with intensive husbandry implicated as a risk factor [47, 48, 51]; 2.3% in United States herds [52]; and variable across South America [37].

In Türkiye, epidemiological data remain limited. Cattle seroprevalence ranges from 4.6% to 18%, with infection in 7 of 15 herds [53–56]; sheep seroprevalence varies from 0% to 29.4% by breed, with an overall mean of 7.3% across 2,257 animals and disease in 10 of 11 herds surveyed [36]. For goats, a Burdur province field study recorded 24% seroprevalence among 150 animals by ELISA [53], and a preliminary investigation at the Bandırma Sheep Breeding and Research Institute identified 13.7% seropositivity (18/131 Saanen goats; unpublished data).

These fragmentary, geographically restricted observations almost certainly underestimate the true MAP burden in Turkish goat populations. Türkiye supports approximately 11.2 million goats [57] across more than ten indigenous breeds adapted to diverse agro-ecological niches from semi-arid Central Anatolian steppes to the Mediterranean and Black Sea littoral. This genetic diversity, combined with the near-complete absence of systematic MAP surveillance, represents a substantial epidemiological knowledge gap and a uniquely valuable resource for investigating breed-level variation in MAP susceptibility. The present study was therefore designed as the first national, multi-breed cross-sectional serosurvey of paratuberculosis in Turkish goat populations, aiming to determine breed- and herd-level seroprevalence, quantify breed-associated risk differentials, and estimate each breed’s descriptive contribution to population-level seroprevalence burden through breed-stratified burden analysis.

## Materials and methods

### Ethics statement

All biological samples were obtained by authorized veterinarians in accordance with the animal welfare regulations in force in Türkiye. Prior to initiating the study, the required approvals were secured from the Ministry of Agriculture and Forestry. After sampling, the animals continued to be housed under routine farm management conditions with standard feeding and care practices, and none of the animals were subjected to euthanasia as part of this study.

### Sample collection, and storage

For each animal, blood samples (approximately 5–6 mL) were collected into 10 ml yellow-capped gel tubes and centrifuged at 5,000 rpm as soon as practicable after collection. The resulting sera were transferred into 2 mL screw-capped tubes and stored at − 20 °C until laboratory analysis.

### Animals, serological testing, and study design

A total of 3,372 goats from 36 herds across 11 provinces were recruited from the National Small Ruminant In Situ Breeding Project and the Domestic Animal Genetic Resources In Situ Conservation Program. MAP seropositivity was evaluated in all serum samples using a commercial indirect ELISA kit (IDvet ID Screen^®^ Paratuberculosis Indirect, Grabels, France) performed in accordance with the manufacturer’s instructions. Optical density was measured at 450 nm, and Sample-to-Positive (S/P%) values were calculated for result interpretation. Assay validity was confirmed when the ratio of the mean S/P value of the negative controls to that of the positive controls was ≤ 1/5. Samples were subsequently classified as negative (S/P% < 60%), suspicious (S/P% 60–70%), or positive (S/P% ≥ 70%).

Dataset refinement proceeded in three sequential steps. First, three animals whose serum samples yielded invalid ELISA results due to excessive hemolysis were excluded, leaving 3,369 successfully tested individuals. Second, white Angora goats (*n* = 301; apparent prevalence = 0.00%) were excluded from all seroprevalence and regression analyses, as universal seronegativity within this breed precluded meaningful comparative analysis; the five herds composed entirely of white Angora goats were likewise removed from herd-level analyses, reducing the dataset to 3,069 animals across 31 herds. This exclusion was applied at the breed level because all five White Angora herds were universally seronegative, eliminating any within-breed variation required for comparative analysis; individual herds from other breeds that recorded zero seropositives (e.g., Herds 11 and 13) were retained because those breeds demonstrated seropositive herds at other locations, preserving the within-breed variability necessary for valid statistical representation. Third, 34 samples with suspicious ELISA results (S/P% 60–70%) were considered analytically uncertain and excluded. All prevalence calculations and statistical analyses were therefore conducted on a final dataset of 3,035 animals from 31 herds. Because enrollment was limited to project-affiliated breeding and conservation herds rather than a random national sample, a degree of selection bias cannot be excluded and should be considered when interpreting the prevalence estimates reported herein.

### Statistical analysis

#### Data management and serological classification

Raw ELISA output was expressed as a sample-to-positive percentage (SP%), calculated according to the manufacturer’s formula (IDvet IDScreen^®^ Paratuberculosis Indirect ELISA). Animals were classified into three serological categories using manufacturer-recommended cut-off values: seronegative (SP% < 60), suspicious (60 ≤ SP% < 70), and seropositive (SP% ≥ 70). Prior to statistical analysis, the dataset underwent a structured quality-control pipeline that included detection of duplicate identifiers, range validation for SP% values, verification of consistency between the ‘Results’ field and derived SP% categories, and enumeration of missing data by variable. Forty-five animals with missing age records (1.48% of the analytical dataset) were retained in regression models as a separate analytical category (“Age NA”) rather than excluded, to preserve the full analytical sample.

#### Descriptive statistics and seroprevalence estimation

Apparent seroprevalence (AP%) was estimated at the animal level as the proportion of seropositive individuals within each stratum (breed, sex, age group, and herd). Exact binomial 95% confidence intervals (CIs) were computed using the Clopper–Pearson method for all prevalence estimates. The Clopper–Pearson interval was chosen over asymptotic alternatives because it preserves the nominal coverage probability for small and moderate cell sizes without requiring a normal approximation. Full formulae for the Clopper–Pearson estimator are provided in the Supplementary Methods. Herd-level seroprevalence was defined as the proportion of herds containing at least one seropositive animal. The continuous distribution of SP% values was characterised using kernel density estimation.

#### Risk factor analysis: prevalence ratio estimation and herd-level clustering assessment

To estimate the association between host-level factors and seropositivity, modified Poisson regression with a log link function was used in preference to logistic regression. In cross-sectional studies with common outcomes (AP% > 10%), odds ratios derived from logistic regression substantially overestimate relative risks; Poisson regression with robust variance estimation directly yields Prevalence Ratios (PRs), which are more interpretable measures of association [58, 59]. All categorical variables were dummy-coded with the breed exhibiting the lowest crude seroprevalence designated as the reference category, and sex and the youngest age group (2–3 years) serving as additional reference levels. PRs and 95% CIs were obtained by exponentiating the regression coefficients and corresponding confidence limits. Statistical significance was assessed using Wald z-statistics, with a two-sided α = 0.05. Full model specification, the sandwich (Huber–White) robust covariance estimator, and IRLS convergence criteria are detailed in the Supplementary Methods.

##### Univariate prevalence ratio analysis (Model A)

Separate univariable Modified Poisson regression models were fitted for each predictor (breed, age group, and sex) independently, without adjustment for other covariates. This yielded unadjusted, crude PRs for each variable, serving as the baseline against which the degree of confounding could be assessed by comparison with Model B.

##### Multivariable prevalence ratio analysis with individual-level robust standard errors (Model B)

Breed, age group, and sex were entered simultaneously into a single Modified Poisson regression model, yielding confounder-adjusted PRs. Robust standard errors were estimated using the individual-level sandwich (Huber–White) covariance estimator, in which each observation constitutes its own cluster. Within-herd correlation of seropositivity was not accounted for at this stage; this limitation is addressed in Model D.

##### Intraclass correlation coefficient estimation (Model C)

Prior to fitting the cluster-robust model, the degree of within-herd clustering of seropositivity was quantified using the Intraclass Correlation Coefficient (ICC), estimated via the one-way random-effects ANOVA method [60]; the full formula is provided in the Supplementary Methods. ICC values < 0.05 were interpreted as negligible clustering, 0.05–0.20 as moderate, and > 0.20 as high. This diagnostic step was used to evaluate whether herd-level clustering was sufficiently substantial to warrant the cluster-robust standard error estimation applied in Model D.

##### Multivariable prevalence ratio analysis with cluster-robust standard errors (Model D)

Model D retained the same fixed-effect covariate structure as Model B (breed, age group, and sex), yielding identical point estimates. Standard errors were, however, estimated using a cluster-robust sandwich covariance matrix with herd as the clustering unit, thereby accounting for the within-herd correlation of seropositivity quantified in Model C. Where ICC exceeded 0.05, Model D is considered the primary inferential model, as its confidence intervals appropriately reflect the hierarchical structure of the data.

#### Age effect analysis

The relationship between age and seropositivity was investigated using two complementary approaches. First, animals were stratified into three ordinal age groups (2–3, 4–5, and ≥ 6 years), and the Cochran–Armitage test for linear trend was applied to evaluate whether the proportion of seropositive animals changed monotonically across age categories. Full test statistic formula is provided in the Supplementary Methods. Second, a quadratic age term was incorporated into an extended Poisson regression model, and locally weighted scatterplot smoothing (LOESS, span = 0.75, tricubic kernel weights) was applied to the continuous age data as a non-parametric reference.

#### Breed-stratified seroprevalence burden analysis

To quantify each breed’s contribution to the overall MAP seroprevalence burden at the population level, a breed-stratified burden metric was calculated using the Population Attributable Fraction (PAF) framework [61]. For exposure categories identified as statistically significant risk factors in the multivariable model, PAF was estimated using the formula of [61]: PAF = pᵉ(PR − 1) / [1 + pᵉ(PR − 1)], where pᵉ is the proportion of the study population belonging to the breed category and PR is the adjusted prevalence ratio from Model B. It is important to note that breed is not a modifiable exposure in the same sense as management risk factors; accordingly, these estimates should be interpreted as descriptive breed-stratified burden indicators rather than as predictions of the reduction in seroprevalence that would result from eliminating a breed. Their value lies in prioritising breeds for targeted surveillance and intervention, not in implying causal exposure removal [61, 62].

#### Statistical power and sample adequacy

A precision-based sample size analysis was conducted. Briefly, the achieved sample size of *n* = 3,035 yields a margin of error of ± 1.24% points at the 95% confidence level, satisfying the ± 2.0%, ± 3.0%, and ± 5.0% precision thresholds.

#### Significance thresholds and software

Statistical significance was defined as *p* < 0.05 throughout. Results are presented as point estimates with 95% CIs. The complete analytical pipeline, including data loading, quality control, modelling, and report generation, was implemented as a custom Python script using NumPy, SciPy, pandas, Matplotlib, and Seaborn.

## Results

### Sample distribution

**Fig. 1:**
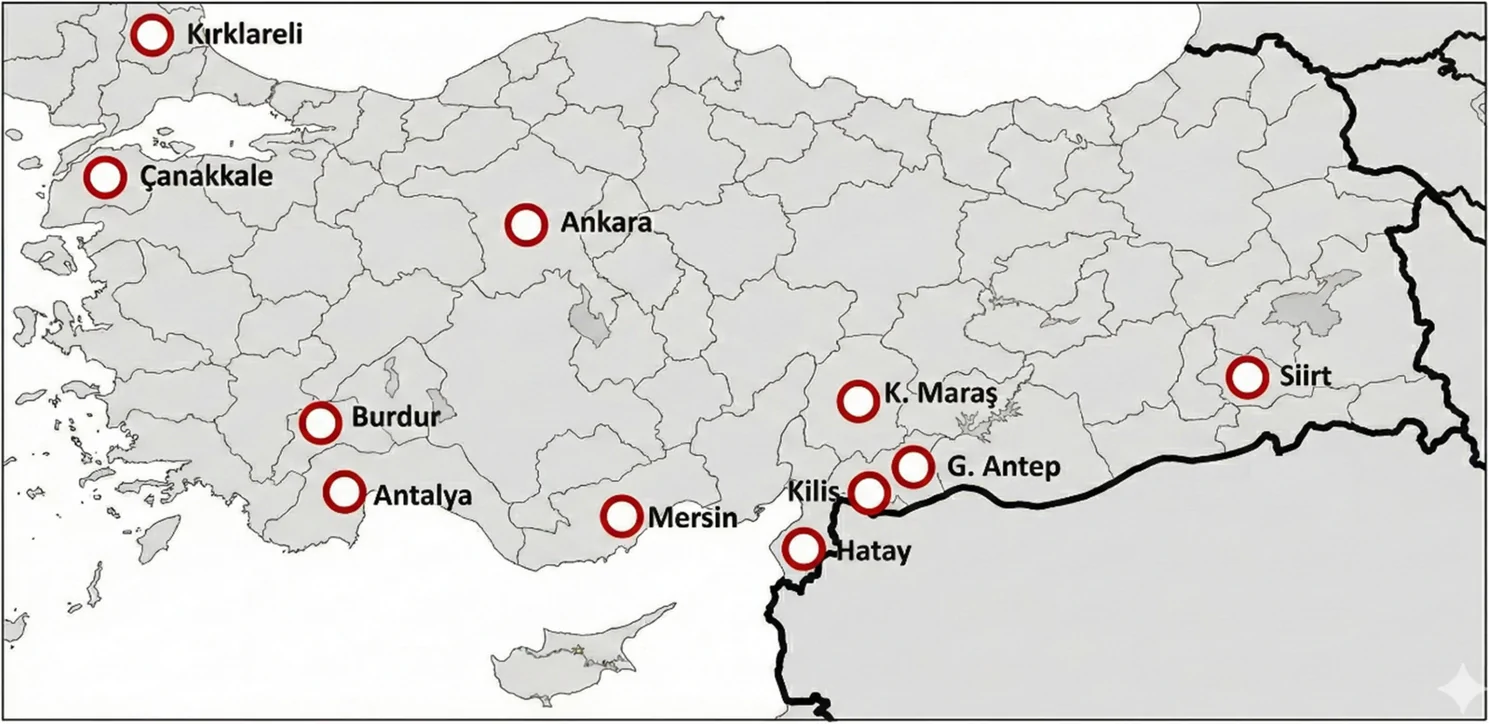
Geographic distribution of sampling sites across 11 provinces located within four regions

The study included goats representing several major breeds raised in different parts of Türkiye and maintained under diverse production systems. Animals were sampled from multiple farms located in provinces representing the Mediterranean, Central Anatolia, Southeastern Anatolia, and Marmara regions. The sampling strategy was designed to capture the geographic and genetic diversity of goat populations included in the study while reflecting the structure of breeding herds used in production systems. Detailed information on the number of animals, sex distribution, farm sources, and geographic origins of the sampled populations is presented in Table 1 and Fig. 1.

**Table 1:** Distribution of sampled goat populations by breed, sex, farm, and geographic origin in Türkiye

Breeds	*n*	Male	Female	Farm	Location (Provinces)	Geographic region
Hairy goat	500	17	483	6	Antalya, Burdur, Kahramanmaraş, Mersin	Mediterranean
Honamlı goat	464	11	453	5	Antalya, Burdur	
Damascus	470	22	448	6	Hatay	
Angora goat (white)	301	22	279	5	Ankara	Central Anatolia
Angora goat (colored)	185	0	185	3	Siirt	Southeastern Anatolia
Kilis goat	499	10	489	4	Gaziantep, Kilis	Southeastern Anatolia
Turkish Saanen	475	6	469	3	Çanakkale	Marmara
Maltese goat	478	8	470	4	Kırklareli	Marmara
Total	**3**,**372**	**96**	**3**,**276**	**36**	**11**	**4**

White Angora goats (*n* = 301) are included in this table as part of the enrolled sampling frame but were excluded from all comparative seroprevalence and regression analyses due to universal seronegativity across all five sampled herds, which precluded meaningful statistical modelling

Throughout the Results section, a single analytical framework is applied. Of the 3,069 successfully tested samples, 34 with suspicious ELISA results were completely excluded from the dataset. Consequently, all analyses in this study were conducted on the remaining 3,035 animals.

### Overall and breed-level seroprevalence

**Fig. 2:**
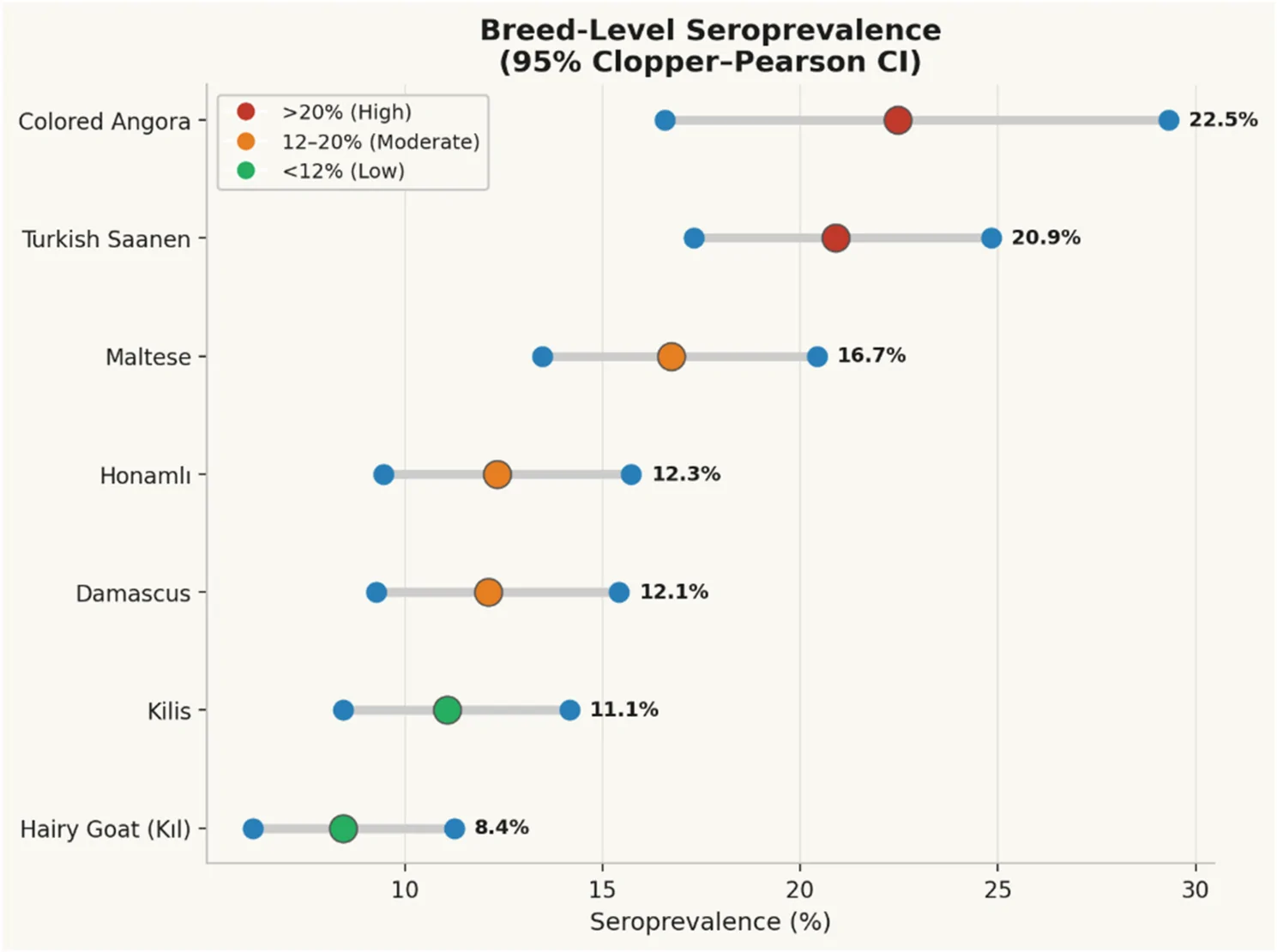
Breed-level apparent seroprevalence with exact 95% Clopper–Pearson confidence intervals (dumbbell plot)

Breed-level apparent seroprevalence ranged from 8.45% to 22.47% across the seven goat breeds included in the analysis (Table 2, Fig. 2). Colored Angora and Turkish Saanen recorded the highest estimates (22.47%, 95% CI: 16.57–29.32; and 20.89%, 95% CI: 17.31–24.83, respectively), both exceeding the 20% threshold designated as high seroprevalence. Maltese fell within the moderate range at 16.74% (95% CI: 13.48–20.42), while Honamlı, Damascus, and Kilis returned closely grouped estimates between 11.07% and 12.33%, each with overlapping confidence intervals. Hairy Goat (Kıl) returned the lowest apparent seroprevalence of 8.45% (95% CI: 6.16–11.25). The width of confidence intervals varied across breeds, with smaller subgroups such as Colored Angora (*n* = 178) yielding notably wider intervals compared to larger breeds such as Kilis and Hairy Goat (*n* = 497 each), reflecting the influence of sample size on estimate precision.

**Table 2:** Breed-level MAP apparent seroprevalence (descriptive framework, *n* = 3,035; Clopper–Pearson 95% CI)

Breed	*n*	Positive	AP%	95% CI Lower	95% CI Upper
Colored Angora	178	40	22.47	16.57	29.32
Turkish Saanen	474	99	20.89	17.31	24.83
Maltese	472	79	16.74	13.48	20.42
Honamlı	454	56	12.33	9.45	15.72
Damascus	463	56	12.1	9.27	15.42
Kilis	497	55	11.07	8.45	14.16
Hairy Goat (Kıl)	497	42	8.45	6.16	11.25
Overall	3,035	427	14.07	12.83	15.31

### Herd-level seroprevalence

**Fig. 3:**
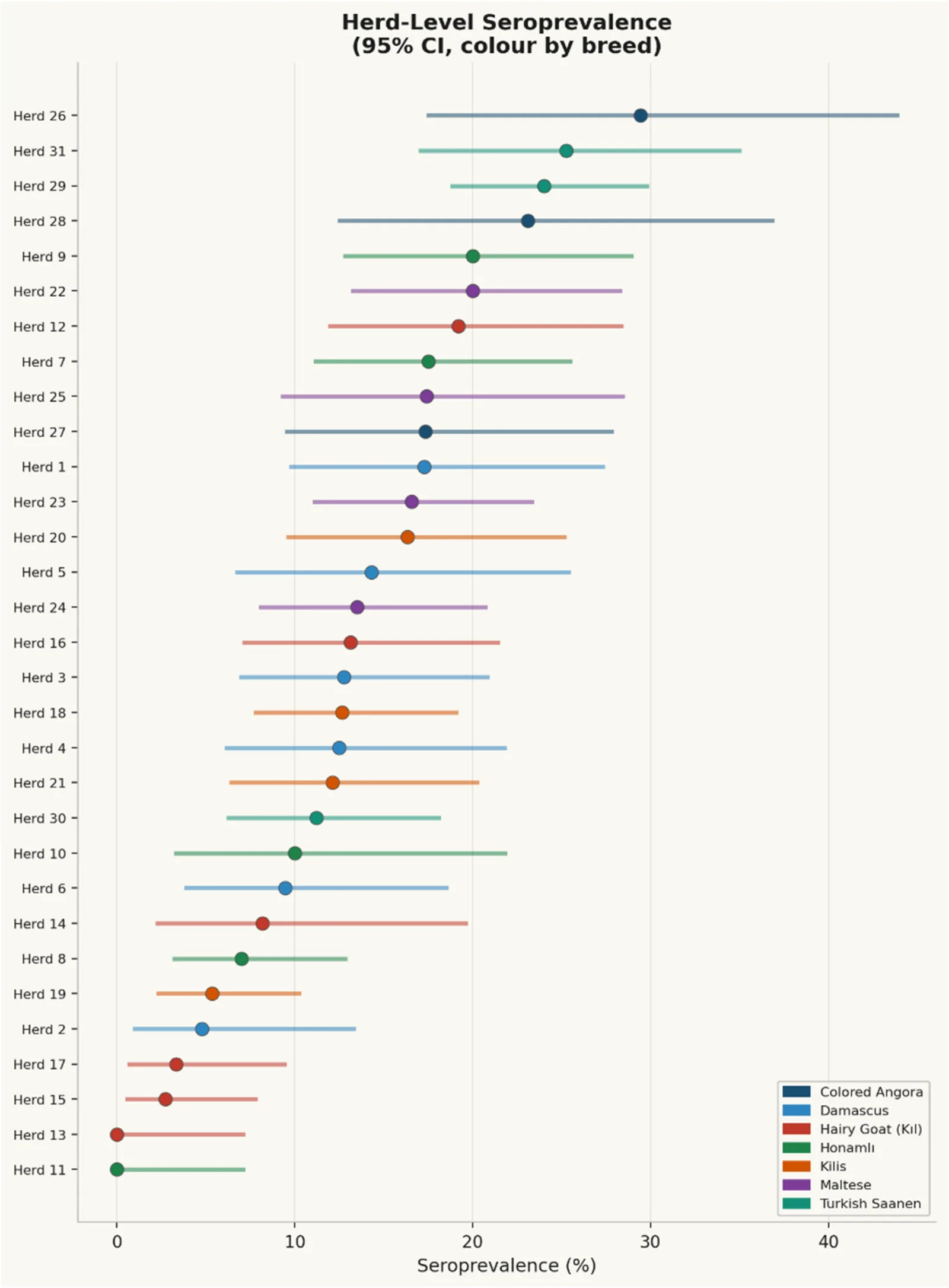
Forest plot of within-herd MAP seroprevalence (31 herds). Point estimates with 95% Clopper–Pearson CIs

Of the 31 herds included in the analysis, 29 (93.5%) contained at least one seropositive animal, indicating near-universal herd-level MAP exposure across the study population. Within-herd apparent seroprevalence ranged widely from 0.00% to 29.41%, reflecting substantial heterogeneity in infection burden across herds (Table 3, Fig. 3). The two herds recording zero seropositives (Herds 11 and 13, both *n* = 50) returned upper confidence interval bounds of 7.11%, consistent with the limited precision inherent to smaller herd sizes rather than definitive evidence of MAP-free status. At the upper end, four herds exceeded 20% apparent seroprevalence — all belonging to either Colored Angora or Turkish Saanen breeds — while the majority of herds (*n* = 17) fell below the 15% threshold. Confidence interval widths varied considerably as a function of herd size, with smaller herds (*n* = 49–63) yielding intervals spanning up to approximately 17–19% points, whereas the largest herds (*n* = 150–250) produced substantially narrower estimates. No single region accounted exclusively for high or low seroprevalence herds, as within-region variability was apparent across multiple locations.

**Table 3:** Herd-level MAP apparent seroprevalence (Clopper–Pearson 95% CI). All 31 herds

Herd	Breed	Region	*n*	Positive	AP%	95% CI Lower	95% CI Upper
26	Colored Angora	Siirt	51	15	29.41	17.49	43.83
31	Turkish Saanen	Çanakkale	99	25	25.25	17.06	34.98
29	Turkish Saanen	Çanakkale	250	60	24	18.84	29.79
28	Colored Angora	Siirt	52	12	23.08	12.53	36.84
9	Honamlı	Burdur	105	21	20	12.83	28.93
22	Maltese	Kırklareli	120	24	20	13.25	28.28
12	Hairy Goat (Kıl)	Burdur	99	19	19.19	11.97	28.34
7	Honamlı	Burdur	120	21	17.5	11.17	25.5
25	Maltese	Kırklareli	69	12	17.39	9.32	28.41
27	Colored Angora	Siirt	75	13	17.33	9.57	27.81
1	Damascus	Hatay	81	14	17.28	9.78	27.3
23	Maltese	Kırklareli	157	26	16.56	11.11	23.32
20	Kilis	Kilis	98	16	16.33	9.63	25.16
5	Damascus	Hatay	63	9	14.29	6.75	25.39
24	Maltese	Kırklareli	126	17	13.49	8.06	20.72
16	Hairy Goat (Kıl)	Mersin	99	13	13.13	7.18	21.41
3	Damascus	Hatay	102	13	12.75	6.96	20.81
18	Kilis	Kilis	150	19	12.67	7.8	19.07
4	Damascus	Hatay	80	10	12.5	6.16	21.79
21	Kilis	G.Antep	99	12	12.12	6.42	20.22
30	Turkish Saanen	Çanakkale	125	14	11.2	6.26	18.08
10	Honamlı	Antalya	50	5	10	3.33	21.81
6	Damascus	Hatay	74	7	9.46	3.89	18.52
14	Hairy Goat (Kıl)	Antalya	49	4	8.16	2.27	19.6
8	Honamlı	Burdur	129	9	6.98	3.24	12.83
19	Kilis	Kilis	150	8	5.33	2.33	10.24
2	Damascus	Hatay	63	3	4.76	0.99	13.29
17	Hairy Goat (Kıl)	Mersin	90	3	3.33	0.69	9.43
15	Hairy Goat (Kıl)	K.Maraş	110	3	2.73	0.57	7.76
13	Hairy Goat (Kıl)	Antalya	50	0	0	0	7.11
11	Honamlı	Antalya	50	0	0	0	7.11

### SP% distribution analysis

**Fig. 4:**
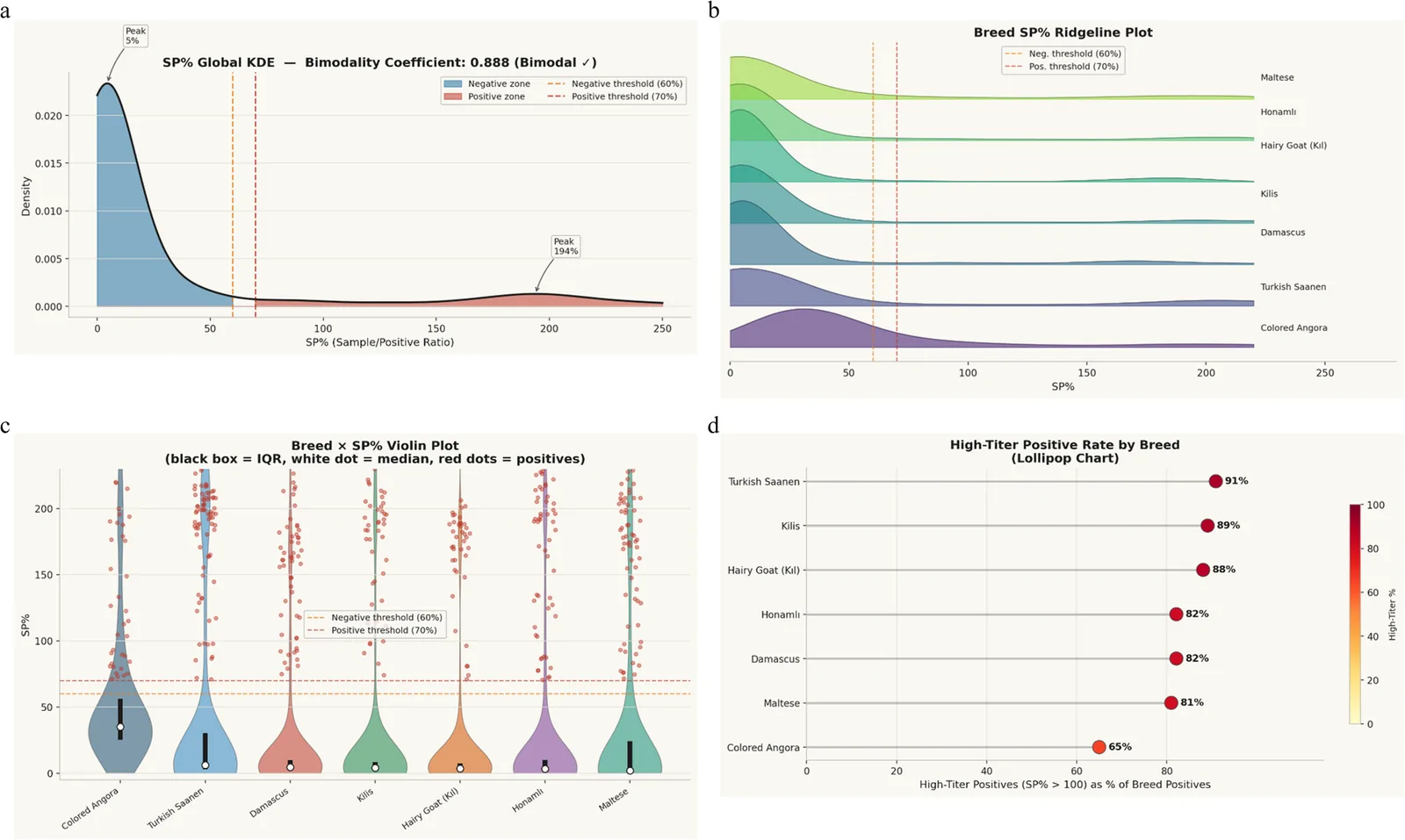
**a**. Zoned kernel density estimate showing negative, suspicious, and positive zones. **b**. Breed-level SP% ridgeline plot. Dashed vertical line marks positivity threshold (SP% = 70). **c**. Violin plots with inner box-and-whisker showing medians and interquartile ranges. **d**. High-titre positive rate (SP% > 100) lollipop chart by breed

The SP% distribution across the overall study population exhibited marked bimodality (bimodality coefficient = 0.888; Fig. 4a), indicating a clear population-level separation between seropositive and seronegative animals. Breed-specific distributional patterns, visualized through ridgeline and violin plots (Fig. 4b and c), revealed considerable heterogeneity in SP% profiles across breeds (Table 4). Colored Angora recorded the highest median SP% (35.14; Q1-Q3: 25.08–56.23) and the highest mean (57.45), with individual values reaching a maximum of 290.35. Turkish Saanen also displayed a comparatively elevated median of 6.17 (Q1-Q3: 3.13–30.51) and a mean of 47.32, alongside a maximum of 304.53. The remaining breeds — Damascus (median: 4.62), Kilis (4.14), Hairy Goat/Kıl (3.52), Honamlı (3.26), and Maltese (2.02) — returned substantially lower medians, though all exhibited wide ranges extending to maximum values between 251.24 and 326.18, indicative of high-titre outliers within otherwise low-SP% populations. Notably, Maltese presented a Q1 of 0.00, reflecting a considerable proportion of animals at or near zero SP%, despite a mean of 35.82 driven by extreme upper-end values. High-titre positivity rates (SP% > 100) varied across breeds, as illustrated in Fig. 4d.

**Table 4:** Breed SP% distribution statistics

Breed	Median	Q1	Q3	Min	Max	Mean
Colored Angora	35.14	25.08	56.23	0.00	290.35	57.45
Damascus	4.62	2.66	9.69	0.00	251.24	24.65
Hairy Goat (Kıl)	3.52	2.31	7.42	0.04	326.18	19.76
Honamlı	3.26	1.36	10.08	0.00	288.79	25.95
Kilis	4.14	2.46	8.42	0.00	268.58	25.93
Maltese	2.02	0.00	24.16	0.00	309.58	35.82
Turkish Saanen	6.17	3.13	30.51	0.00	304.53	47.32

### Effect of age and sex

Seroprevalence estimates across the three age groups (2–3, 4–5, and ≥ 6 years) were closely grouped, ranging from 12.37% to 15.26%, with overlapping confidence intervals throughout (Table 5). Neither the Cochran–Armitage trend test nor the likelihood-ratio test for the quadratic age term returned statistically significant results (*p* = 0.796 and *p* = 0.109, respectively). The substantially wider confidence interval observed for males reflected the considerable imbalance in group sizes (*n* = 74 vs. *n* = 2,961) rather than a true divergence in seroprevalence.

**Table 5:** Age group and sex-based seroprevalence

Variable	*n*	Positive	AP%	95% CI Low	95% CI High
Age 2–3 yr	1,147	155	13.51	11.59	15.63
Age 4–5 yr	1,350	206	15.26	13.38	17.29
Age ≥ 6 yr	493	61	12.37	9.6	15.61
Male	74	12	16.22	8.67	26.61
Female	2,961	415	14.02	12.78	15.32

Cochran–Armitage: z = −0.258, *p* = 0.796. LRT (quadratic): χ² = 2.567, *p* = 0.109. Model B PR for female: 0.692 (*p* = 0.177)

### Univariate prevalence ratio analysis (Model A)

**Fig. 5:**
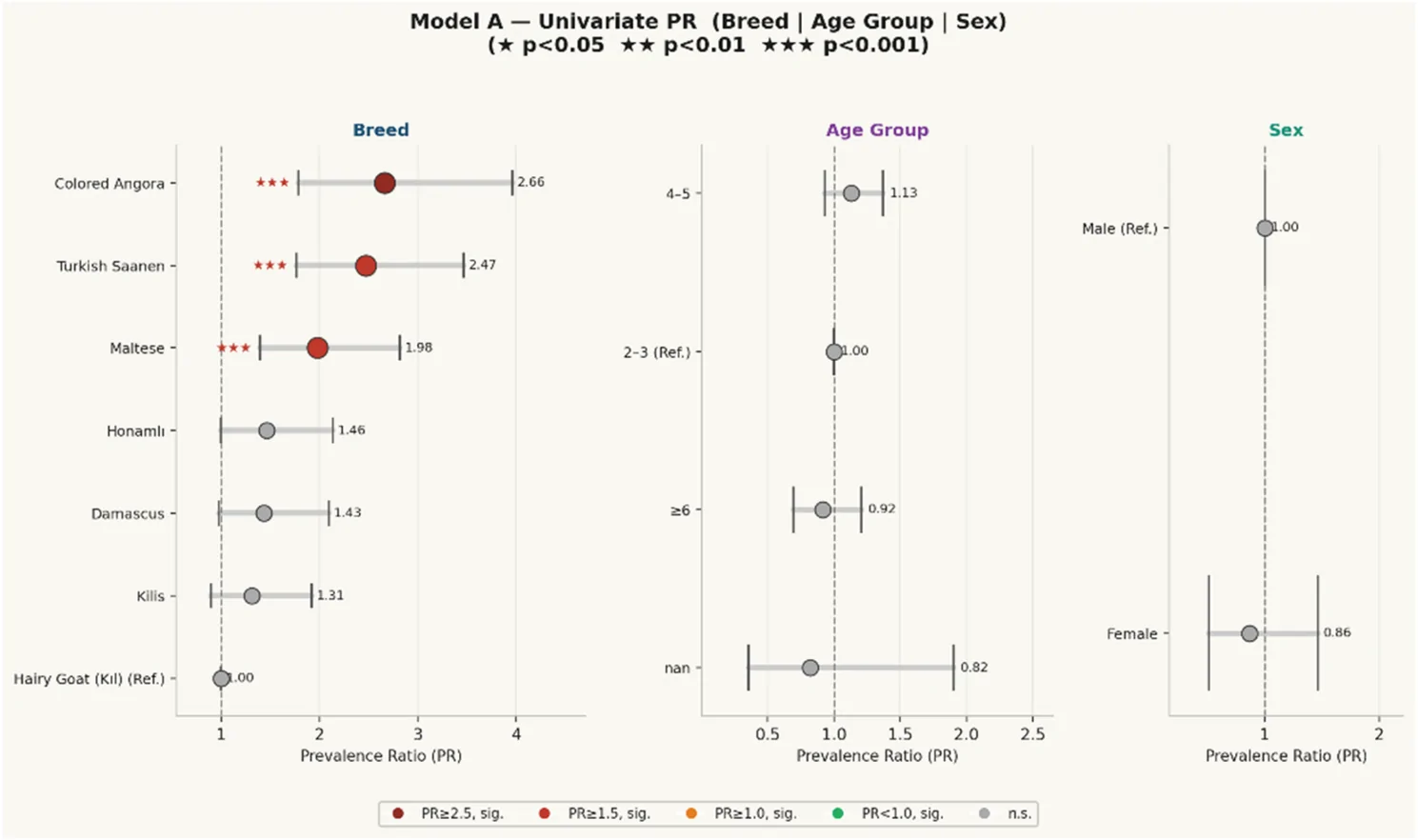
Unadjusted prevalence ratios with 95% confidence intervals for breed, age group, and sex from univariate Modified Poisson regression (Model A)

Univariate modified Poisson regression (Model A) identified breed as the sole predictor with statistically significant associations with paratuberculosis seropositivity, while age group and sex yielded no significant effects in unadjusted analyses (Fig. 5, Table 6). Among the seven breeds assessed relative to Hairy Goat as the reference, Colored Angora and Turkish Saanen exhibited the highest estimated prevalence ratios (PR = 2.66, 95% CI: 1.79–3.96, *p* < 0.0001; and PR = 2.47, 95% CI: 1.76–3.47, *p* < 0.0001, respectively), followed by Maltese (PR = 1.98, 95% CI: 1.39–2.82, *p* = 0.0001). The remaining breeds — Damascus, Honamlı, and Kilis — returned point estimates ranging from 1.31 to 1.46, none of which reached conventional significance thresholds (all *p* > 0.05). The precision of breed-level estimates, as reflected by confidence interval width, was notably narrower for the three significant breeds compared to their non-significant counterparts, a pattern attributable in part to differences in within-breed sample sizes. Neither age group — with 4–5 and ≥ 6 year categories yielding PR estimates of 1.13 and 0.92, respectively, relative to the 2–3 year reference — nor sex (Female vs. Male: PR = 0.86, 95% CI: 0.51–1.46) was associated with seropositivity at any conventional alpha level. The wide confidence intervals observed for the unrecorded age category (nan: PR = 0.82, 95% CI: 0.36–1.90) reflect the limited number of animals with missing age records and preclude meaningful interpretation of that stratum. Collectively, Model A results indicate that breed-level variation constitutes the primary source of unadjusted heterogeneity in seroprevalence across the study population.

**Table 6:** Model A — Univariate Modified Poisson Regression Analysis: Prevalence Ratios for Paratuberculosis Seropositivity

Variable	β	SE	PR	95% CI Low	95% CI High	z	*p*-value
Breeds							
Colored	0.9780	0.2029	2.6590	1.7870	3.9580	4.8200	< 0.0001
Damascus	0.3585	0.1936	1.4310	0.9790	2.0920	1.8520	0.0641
Honamlı	0.3782	0.1935	1.4600	0.9990	2.1330	1.9540	0.0507
Kilis	0.2697	0.1949	1.3100	0.8940	1.9190	1.3840	0.1664
Maltese	0.6834	0.1798	1.9810	1.3920	2.8170	3.8000	0.0001
Turkish Saanen	0.9048	0.1726	2.4720	1.7620	3.4660	5.2430	< 0.0001
Age groups							
4–5	0.1215	0.0985	1.1290	0.9310	1.3700	1.2340	0.2172
≥ 6	-0.0882	0.1412	0.9160	0.6940	1.2080	-0.6240	0.5325
nan	-0.1957	0.4282	0.8220	0.3550	1.9030	-0.4570	0.6476
Sex							
Female	-0.1458	0.2681	0.8640	0.5110	1.4620	-0.5440	0.5865

Reference categories: Breed = Hairy Goat; Age group = 2–3 years; Sex = Male. Model A is a univariate Modified Poisson regression model with robust sandwich standard errors, in which each predictor (breed, age group, and sex) was modelled separately without adjustment for other covariates, yielding unadjusted prevalence ratio estimates

*β* regression coefficient, *SE* robust standard error, *PR* prevalence ratio, *CI* confidence interval, *z* Wald z-statistic

### Multivariable prevalence ratio analysis with individual-level robust standard errors (Model B)

**Fig. 6:**
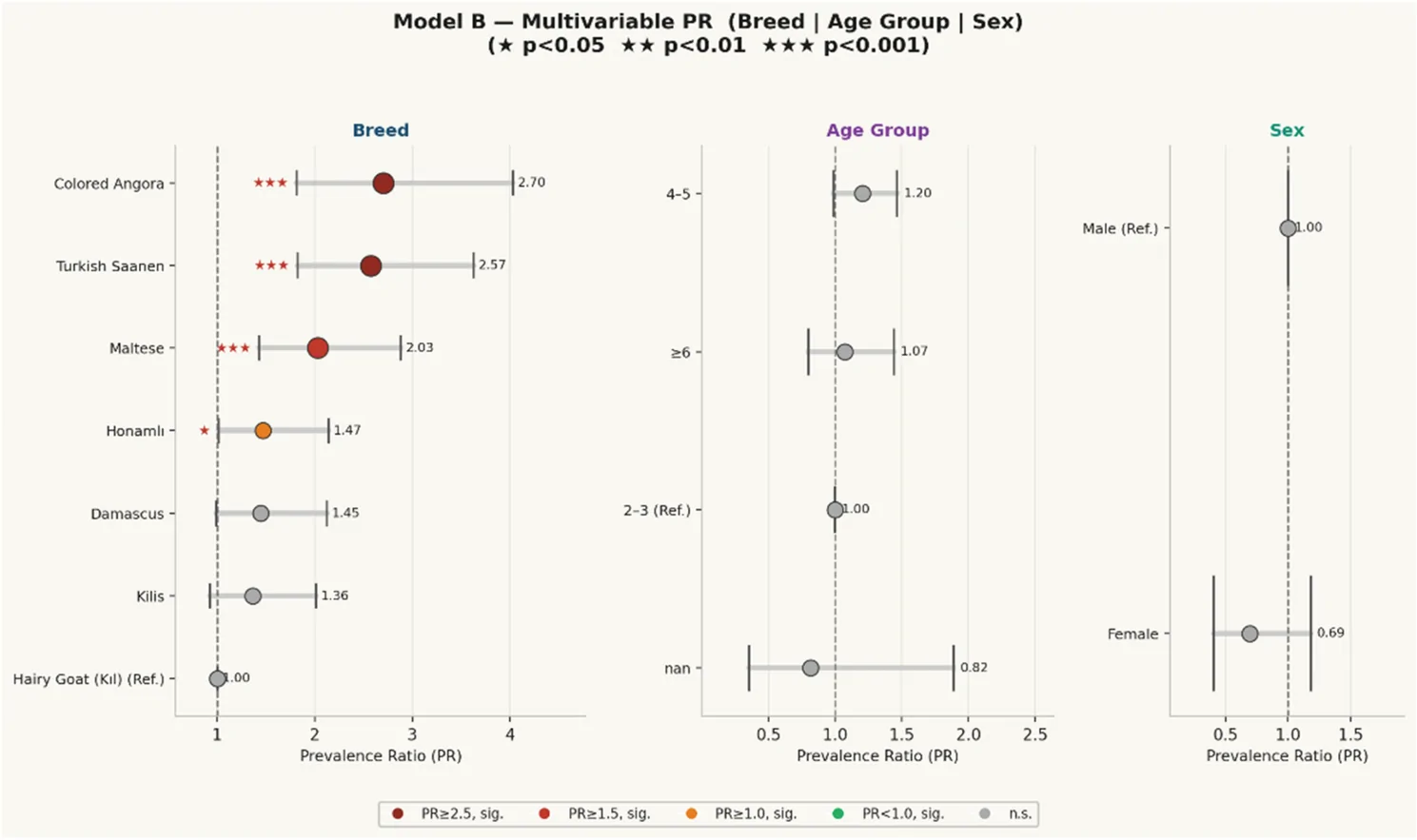
Adjusted prevalence ratios with 95% confidence intervals for breed, age group, and sex from multivariable Modified Poisson regression (Model B)

After simultaneous adjustment for breed, age group, and sex in the multivariable model (Model B), the pattern of associations observed in unadjusted analyses was largely preserved, with breed remaining the only predictor class to yield statistically significant adjusted prevalence ratio estimates (Fig. 6, Table 7). Colored Angora and Turkish Saanen retained the strongest associations with seropositivity relative to Hairy Goat, with adjusted PRs of 2.70 (95% CI: 1.81–4.03, *p* < 0.0001) and 2.57 (95% CI: 1.82–3.62, *p* < 0.0001), respectively, and Maltese followed as the third significant breed (adjusted PR = 2.03, 95% CI: 1.43–2.88, *p* = 0.0001). Notably, Honamlı crossed the α = 0.05 threshold in the adjusted model (PR = 1.47, 95% CI: 1.01–2.14, *p* = 0.0454), a marginal finding absent in Model A, whereas Damascus and Kilis remained non-significant with overlapping confidence intervals that included unity. Comparison of unadjusted and adjusted point estimates across all breeds revealed minimal attenuation — the largest absolute shift was approximately 0.09 PR units for Turkish Saanen — suggesting that age group and sex introduced negligible confounding of the breed–seropositivity relationship. Within the age group domain, the 4–5 year stratum yielded an adjusted PR of 1.20 (95% CI: 0.99–1.47, *p* = 0.065), which approached but did not attain significance, while the ≥ 6 year category and the missing-age stratum produced estimates statistically indistinguishable from the 2–3 year reference. The adjusted PR for females relative to males was 0.69 (95% CI: 0.41–1.18, *p* = 0.177), a direction consistent with Model A but again non-significant and accompanied by wide confidence intervals reflecting the modest female sub-sample. Overall, Model B results corroborate that breed identity is the dominant structured source of variation in paratuberculosis seroprevalence within this population, independent of the other covariates assessed.

**Table 7:** Model B — Multivariable Modified Poisson Regression Analysis: Adjusted Prevalence Ratios for Paratuberculosis Seropositivity

Variable	β	SE	PR	95% CI Low	95% CI High	z	*p*-value
Breeds							
Colored Angora	0.9938	0.2042	2.7020	1.8110	4.0310	4.8680	< 0.0001
Damascus	0.3682	0.1959	1.4450	0.9840	2.1220	1.8790	0.0602
Honamlı	0.3845	0.1921	1.4690	1.0080	2.1410	2.0010	0.0454
Kilis	0.3092	0.1986	1.3620	0.9230	2.0110	1.5570	0.1195
Maltese	0.7067	0.1792	2.0270	1.4270	2.8800	3.9440	0.0001
Turkish Saanen	0.9441	0.1753	2.5710	1.8230	3.6240	5.3870	< 0.0001
Age groups							
4–5	0.1852	0.1005	1.2030	0.9880	1.4650	1.8430	0.0653
≥ 6	0.0704	0.1498	1.0730	0.8000	1.4390	0.4700	0.6384
nan	-0.2032	0.4281	0.8160	0.3530	1.8890	-0.4750	0.6350
Sex							
Female	-0.3675	0.2721	0.6920	0.4060	1.1800	-1.3500	0.1769

Reference categories: Breed = Hairy Goat; Age group = 2–3 years; Sex = Male.  Model B is a multivariable Modified Poisson regression model with robust sandwich standard errors, in which breed, age group, and sex were modelled simultaneously, yielding confounder-adjusted prevalence ratio estimates

*β* regression coefficient, *SE* robust standard error, *PR* prevalence ratio, *CI* confidence interval, *z* Wald z-statistic

### Intraclass correlation coefficient estimation (Model C)

The intraclass correlation coefficient (ICC) for Model C at the herd level was 0.0305, indicating low within-herd clustering (ICC < 0.05). This value suggests that only 3.05% of the residual variance in the outcome was attributable to between-herd differences, implying that the inclusion of covariates in Model C substantially accounted for the clustering structure of the data and that herd-level random effects contributed minimally to the overall variability in the final model.

### Multivariable prevalence ratio analysis with cluster-robust standard errors (Model D)

**Fig. 7:**
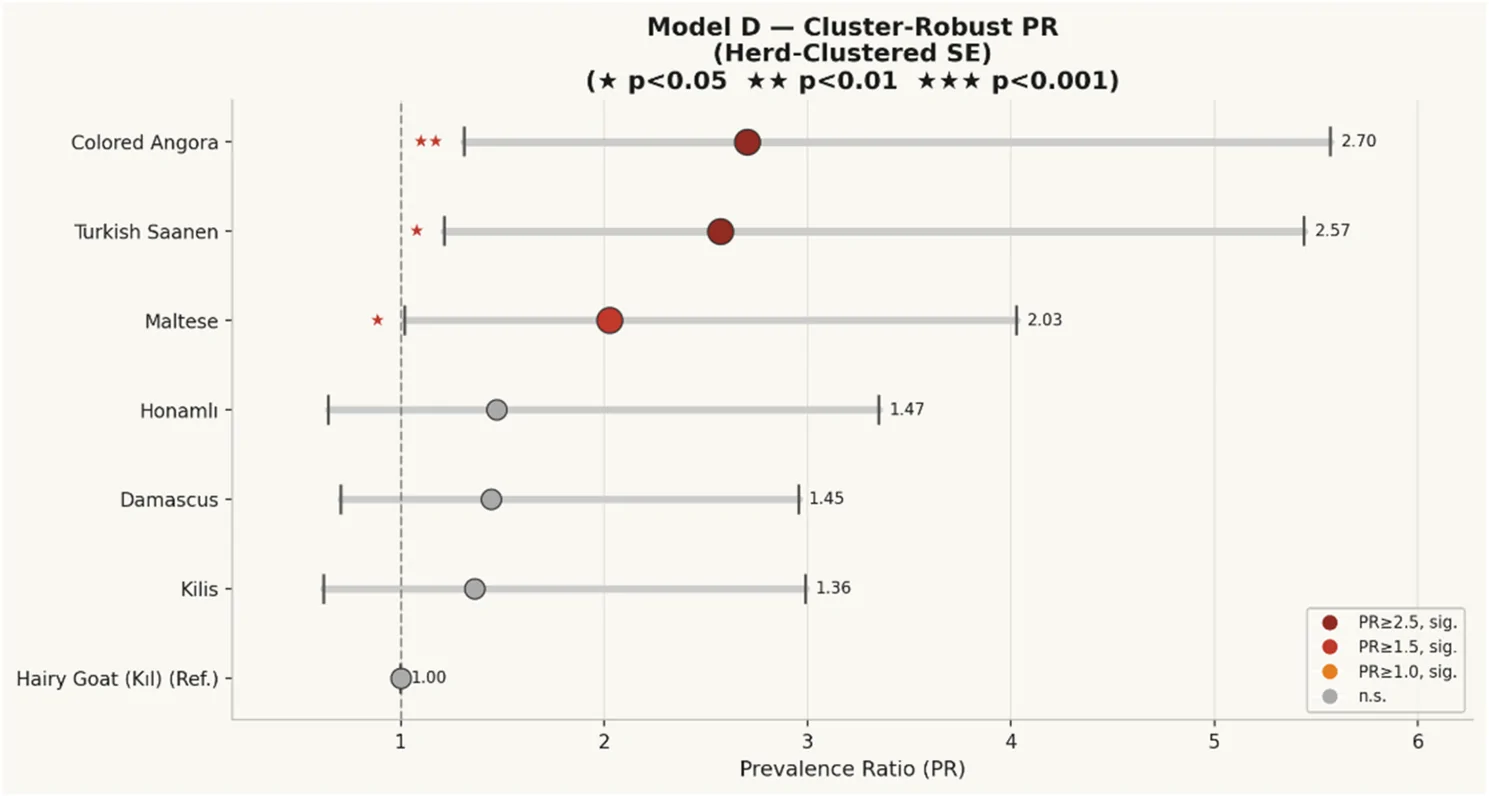
Adjusted prevalence ratios with 95% confidence intervals for breed, age group, and sex from cluster-robust Modified Poisson regression (Model D). Standard errors were clustered by herd. Reference categories: Hairy Goat (breed), 2–3 years (age group), and male (sex)

In the cluster-robust modified Poisson regression (Model D), three breeds returned statistically significant adjusted prevalence ratios relative to the Hairy Goat reference category: Colored Angora (PR = 2.702; 95% CI: 1.310–5.570; *p* = 0.007), Turkish Saanen (PR = 2.571; 95% CI: 1.214–5.442; *p* = 0.014), and Maltese (PR = 2.027; 95% CI: 1.021–4.027; *p* = 0.044; Table 8, Fig. 7). The remaining breeds — Damascus, Honamlı, and Kilis — yielded elevated point estimates but wide confidence intervals crossing unity, and none reached statistical significance (*p* > 0.31). Neither age group differed significantly from the 2–3 year reference (*p* = 0.163 and *p* = 0.734 for the 4–5 and ≥ 6 year groups, respectively), nor did sex emerge as a significant predictor (PR = 0.692 for females; *p* = 0.157). Standard errors in Model D were estimated using a cluster-robust sandwich estimator with herd as the clustering unit, yielding identical point estimates to Model B while accounting for within-herd correlation of seropositivity.

**Table 8:** Model D — Cluster-Robust Modified Poisson Regression Analysis: Adjusted Prevalence Ratios for Paratuberculosis Seropositivity

Variable	β	SE	PR	95% CI Low	95% CI High	z	*p*-value
Breeds							
Colored Angora	0.9938	0.3691	2.7020	1.3100	5.5700	2.6920	0.0071
Damascus	0.3682	0.3654	1.4450	0.7060	2.9570	1.0080	0.3135
Honamlı	0.3845	0.4208	1.4690	0.6440	3.3510	0.9140	0.3608
Kilis	0.3092	0.4011	1.3620	0.6210	2.9910	0.7710	0.4408
Maltese	0.7067	0.3502	2.0270	1.0210	4.0270	2.0180	0.0436
Turkish Saanen	0.9441	0.3827	2.5710	1.2140	5.4420	2.4670	0.0136
Age groups							
4–5	0.1852	0.1329	1.2030	0.9280	1.5610	1.3940	0.1634
≥ 6	0.0704	0.2068	1.0730	0.7150	1.6090	0.3400	0.7336
nan	-0.2032	0.3325	0.8160	0.4250	1.5660	-0.6110	0.5410
Sex							
Female	-0.3675	0.2599	0.6920	0.4160	1.1520	-1.4140	0.1574

Reference categories: Breed = Hairy Goat; Age group = 2–3 years; Sex = Male.  Model D is a multivariable Modified Poisson regression model in which standard errors were estimated using a cluster-robust sandwich estimator with herd as the clustering unit, accounting for within-herd correlation of seropositivity while yielding the same point estimates as Model B

*β* regression coefficient, *SE* robust standard error, *PR* prevalence ratio, *CI* confidence interval, *z* Wald z-statistic

### Breed-stratified seroprevalence burden estimates

Breed-stratified burden analysis, conducted within the PAF framework and interpreted as descriptive estimates rather than causal attributable fractions (see "Breed-stratified seroprevalence burden analysis" section), indicated that Turkish Saanen accounted for the largest proportional contribution to population-level MAP seroprevalence burden (PAF = 19.70%), followed by Maltese (13.77%) and Colored Angora (9.08%; Table 9). Collectively, the four breeds returning statistically significant adjusted prevalence ratios in Model B (Turkish Saanen, Maltese, Colored Angora, and Honamlı) accounted for approximately 49.11% of the breed-stratified burden. Damascus and Kilis returned non-significant prevalence ratios (*p* = 0.060 and *p* = 0.120, respectively) and their PAF estimates (6.36% and 5.60%) should therefore be interpreted with particular caution. As breed is not a modifiable exposure in the epidemiological sense, these figures are not intended to imply that removal or replacement of any breed would yield a commensurate reduction in seroprevalence; rather, they serve as population-level indicators for prioritising breed-targeted surveillance and control efforts.

**Table 9:** Breed-level †PAF from Model B. Reference: Hairy Goat

Breed	*n*	p_exposed	PR (Model B)	*p*-value	PAF%
Turkish Saanen	474	0.1562	2.571	< 0.0001	19.70
Maltese	472	0.1555	2.027	0.0001	13.77
Colored Angora	178	0.0586	2.702	< 0.0001	9.08
Honamlı	454	0.1496	1.469	0.0454	6.56
Damascus	463	0.1526	1.445	0.0602	6.36*
Kilis	497	0.1638	1.362	0.1195	5.60*

* Not significant in Model B

### Precision and sample adequacy

**Fig. 8:**
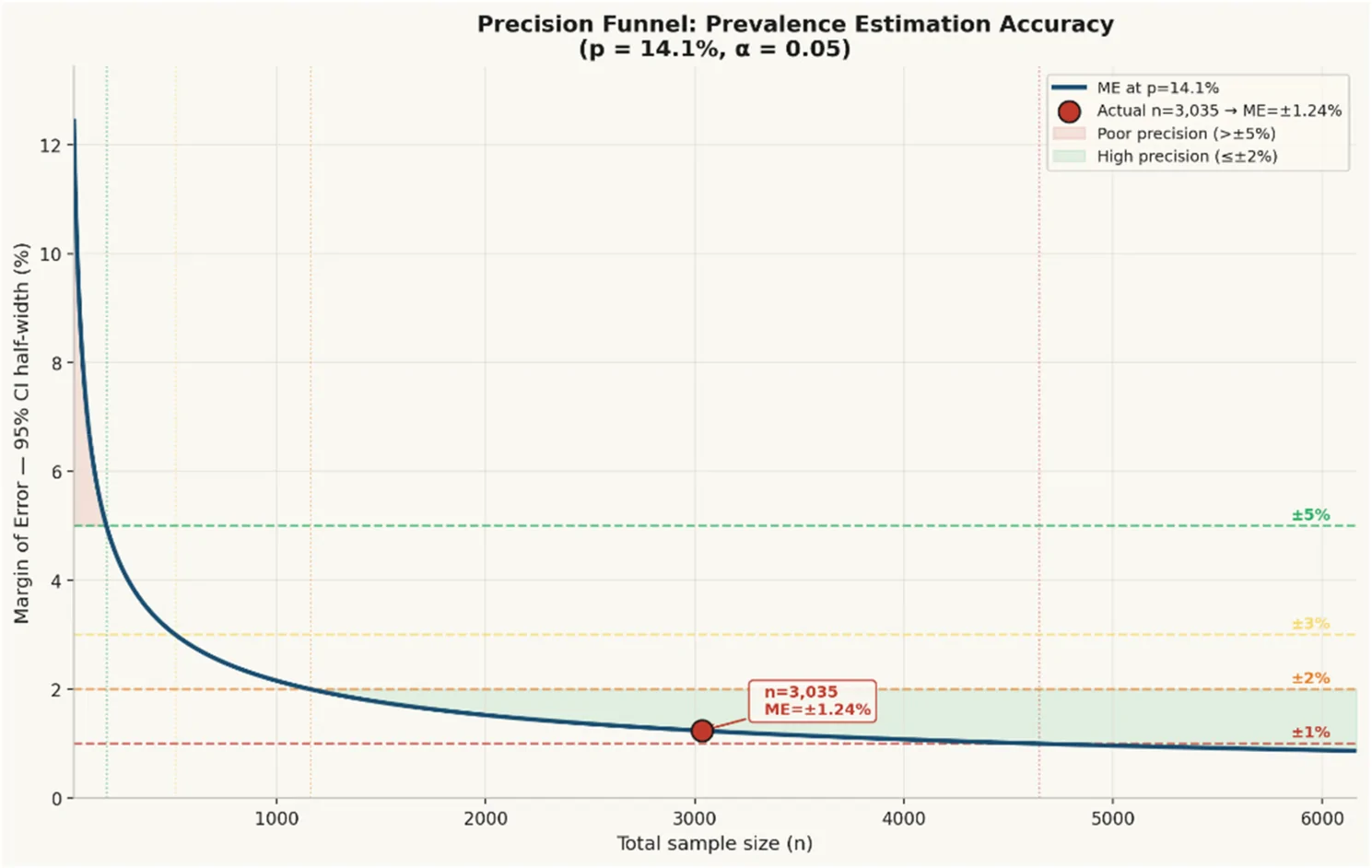
Precision funnel for prevalence estimation showing the current study position relative to precision thresholds

To evaluate the statistical reliability of the prevalence estimates, a precision analysis was conducted based on the final analytical dataset. The margin of error (ME) for the estimated prevalence was calculated at a 95% confidence level. The results indicated that the available sample size yielded a margin of error of ± 1.24%, demonstrating a high level of precision for the overall prevalence estimate (Fig. 8).

## Discussion

This study constitutes the first national, multi-breed seroprevalence survey of paratuberculosis in Turkish goat populations, and one of the largest caprine MAP serosurveys conducted globally. The overall AP of 14.07% and herd-level seropositivity of 93.5% indicate widespread MAP infection across Turkish goat farming systems of diverse geographic, climatic, and operational character. These findings carry immediate implications for veterinary policy, livestock economics, and future research, while raising fundamental questions about the nature of breed-level variation in infection outcome.

### Contextualising the overall seroprevalence

The individual-level seroprevalence of 14.07% detected in the present study must be interpreted through the lens of diagnostic test characteristics and between-study methodological heterogeneity. Positioning this figure within the international literature reveals considerable geographic variation in MAP burden among goat populations. The Turkish estimate clearly exceeds the 2.0% apparent animal-level seroprevalence (true prevalence 3.5%) recorded in Austrian goat herds [47], the 7.4% mean intra-herd seroprevalence documented across 33 northern Italian dairy goat farms [63], and the 5.1% animal-level prevalence observed in Sicilian goat farms [64]. It is broadly comparable to the 12.8% (95% CI: 10.8–15.1%) animal-level seroprevalence reported in northern Portuguese goat herds [51], yet falls below the 20.0% (95% CI: 18.2–21.8%) seroprevalence detected among goats in southern Spain [65] and the 23.9% seropositivity recorded in Indian goats [66]. At the lower end of the spectrum, the true animal-level prevalence in Missouri Boer goat herds was estimated at only 1.4% via the Rogan-Gladen estimator [67], a discordance that powerfully illustrates how low within-herd detection rates can coexist with extensive geographic dissemination of infection.

The divergence between animal-level and herd-level prevalence estimates across studies carries critical epidemiological implications. The recurrent pattern—modest individual seroprevalence paired with high herd positivity, as seen in Sicily, Portugal, and Missouri—suggests that MAP circulates widely through livestock systems even when the proportion of overtly seropositive animals within any given herd remains modest. A key consideration in interpreting all ELISA-based seroprevalence estimates, including the present one, is the well-documented stage-dependent sensitivity of serological testing for MAP. ELISA detects a humoral immune response that typically becomes prominent only in later stages of infection, with reported sensitivities as low as 15% in subclinically infected animals rising to approximately 87% in clinical cases [68]. In caprine populations specifically, serum ELISA sensitivity relative to fecal culture has been reported at 74.3% [69] and milk ELISA sensitivity at 58.4–64.7% [70]. Accordingly, the observed AP of 14.07% likely underestimates the true infection burden, as a substantial proportion of subclinically infected but seronegative animals would not be detected by this approach [71]. Between-study comparability is further constrained by differences in ELISA kit, antigen preparation, cut-off criteria, breed composition, and management intensity.

The sensitivity analysis confirmed that the 34 suspicious classifications had a moderate overall impact (Δ = 1.11% points between Scenarios A and B), though the breed-level impact was notably uneven: Colored Angora showed a 3.79-point shift, suggesting a higher proportion of animals in this breed mount antibody responses that fall near the diagnostic boundary— a pattern that may reflect quantitative differences in antibody dynamics in this breed, though breed-specific ELISA validation data are not available to confirm this interpretation. The precision analysis, with an achieved ME of ± 1.24%, provides the methodological assurance that the overall prevalence estimate constitutes a statistically precise parameter suitable as a baseline for future longitudinal surveillance.

### Near-universal herd contamination and its interpretive consequences

The herd-level seropositivity of 93.5% is perhaps the most epidemiologically consequential finding of this survey. This figure substantially exceeds the already high estimates reported from comparable caprine serosurveys: 90.0% in southern Spain [65], 60.8% in Sicily [64], 60.7% in northern Portugal [51], 58% in northern Italy [63], 54.7% in Missouri Boer goat herds [67], and 50% in Chilean dairy goat herds [69]. The stark contrast with the 11.1% recorded in intensively managed Austrian herds operating under structured biosecurity frameworks [47] further underscores that management intensity and systematic control programmes are the principal determinants of herd-level containment. Collectively, these comparisons reveal a consistent epidemiological pattern across Mediterranean, Near Eastern, and pastoral production systems: once MAP is introduced into a caprine herd, it establishes and persists with remarkable tenacity, with herd-level eradication remaining elusive in the absence of targeted interventions. The forest plot (Fig. 3) vividly illustrates the spectrum from zero to 29.41% within-herd apparent prevalence, with the majority of herds clustering between 5% and 25%. The two seronegative herds (50 animals each) cannot confidently be classified as MAP-free; at this sample size, the upper 95% confidence limit of 7.11% means that within-herd prevalences of several percent cannot be excluded (Table 3).

The ICC of 0.0305 adds a critical interpretive dimension. Despite near-universal herd contamination, within-herd clustering of individual serostatus was remarkably low: only 3.05% of total variance was attributable to herd membership. This suggests that, in this dataset, measurable herd-level clustering of individual serostatus was limited, and that variation in individual seroconversion outcome may not be fully explained by the shared herd environment alone. Whether breed-associated biological differences—immunological, physiological, or genetic—contribute to this pattern is a hypothesis that warrants investigation in future longitudinal and experimental studies; breed-correlated confounders such as regional management traditions, nutritional regimes, or parasite co-infection burden cannot be excluded from cross-sectional data alone. This distinction is an important area for future investigation.

### Breed-level risk hierarchy: evidence and interpretation

The breed-level risk hierarchy (Table 2; Fig. 2) constitutes the analytically richest dimension of the dataset. The 2.66-fold gradient from 8.45% (Hairy Goat) to 22.47% (Colored Angora), translated through modified Poisson regression into adjusted PRs ranging from 1.362 to 2.702 (Table 7), suggests breed-level differences in observed seroprevalence. The choice of modified Poisson regression over logistic regression was analytically necessary: at an overall prevalence of approximately 14%, logistic regression would yield odds ratios that overestimate the true association magnitude by approximately 15–30% for the higher-risk breeds [58, 72], an inflation that could meaningfully distort the epidemiological interpretation.

Notably, four breeds achieved statistical significance in Model B—Colored Angora, Turkish Saanen, Maltese, and Honamlı—a broader pattern of differentiation than typically observed in single-site serosurveys (Table 7; Fig. 6). The emergence of Turkish Saanen as the breed with the second-highest PR (2.571) is particularly noteworthy given its expanding role in the Turkish dairy goat sector and its role as a genetic bridge between European dairy germplasm and local production systems.

In Model D (Table 8; Fig. 7), three breeds maintained significance after conservative cluster-robust correction: Colored Angora (*p* = 0.007), Turkish Saanen (*p* = 0.014), and Maltese (*p* = 0.044). Honamlı, while significant in Model B (*p* = 0.045), lost significance under cluster-robust estimation (*p* = 0.361), attributable to the limited number of herds (*n* = 5) sampled for this breed rather than to a genuine attenuation of the underlying effect. The consistency scatter demonstrates that point estimates are identical across models—only confidence intervals widen—confirming that the low ICC reflects genuine absence of meaningful herd-level confounding of breed effects.

The SP% distribution analysis enriches this picture beyond binary positive/negative classification. Colored Angora not only harbours the highest proportion of seropositives but also the highest median SP% (35.14), the highest mean (57.45), and the greatest density of high-titre individuals (Fig. 4b–d; Table 4). Turkish Saanen follows with a mean SP% of 47.32. This quantitative gradient in antibody loading is an observation that warrants investigation in controlled longitudinal or experimental settings to determine whether it reflects higher within-host pathogen burden, differences in immune interaction with MAP, or breed-correlated environmental and management factors.

### The angora dichotomy: an open question

White Angora goats (*n* = 301 animals with valid ELISA results, sampled exclusively from Ankara Province) were excluded from all comparative seroprevalence and regression analyses on methodological grounds: every animal in this group tested seronegative, yielding an AP of 0.00% with no variance and precluding meaningful statistical modelling or comparison (see "Animals, serological testing, and study design" section for exclusion criteria). This stands in contrast to breeds such as Hairy Goat, which were retained despite including some entirely seronegative herds, because other herds of that breed tested positive—providing the within-breed variation necessary for valid statistical representation. The complete seronegativity of the White Angora group, by contrast, makes it statistically indeterminate rather than demonstrably resistant, and no causal inference about MAP susceptibility can be drawn from these cross-sectional serological data. Yet this complete seronegativity—set against an AP of 22.47% in Colored Angora—constitutes the most striking observational contrast in the dataset and warrants dedicated interpretive attention (Fig. 2).

Several non-mutually exclusive explanations may account for the complete seronegativity of White Angora, and the available data do not allow their relative contributions to be distinguished. A parsimonious explanation is that White Angora herds were never meaningfully exposed to the pathogen. Sampled exclusively from Ankara Province on the semi-arid Central Anatolian plateau, these animals inhabit a harsh continental climate characterised by low humidity, extreme temperature fluctuations, and high ultraviolet radiation—conditions that may substantially curtail MAP environmental survival and limit the establishment of sustained transmission cycles. Colored Angora herds in Siirt Province, by contrast, occupy the warmer, more humid environment of south-eastern Anatolia, where conditions may be considerably more conducive to MAP persistence. Differences in management practices, animal movement histories, herd density, and geographic isolation may further contribute to differential exposure pressure. Additionally, potential differences in ELISA performance across breeds with differing immune response profiles cannot be ruled out without breed-specific test validation data. When the distinct selection histories, geographic isolation, management traditions, and ecological pressures operating on these two populations are considered together, it becomes clear that cross-sectional serological data alone cannot disentangle the relative contributions of differential exposure, differential management, and differential susceptibility.

The statistical boundary of this contrast is nonetheless informative. The upper 95% confidence limit for White Angora (1.22%) means that a true prevalence of up to approximately 1% cannot be excluded—yet even this upper bound would represent an eighteen-fold differential relative to Colored Angora, a magnitude that demands explanation regardless of its ultimate cause. Accordingly, no causal conclusions about breed-level resistance can be drawn from this study, and this natural contrast is identified as a compelling starting point for future controlled investigations, including experimental challenge studies under standardised exposure conditions and comparative genomic analyses designed to reveal whether the two sub-varieties harbour distinct allelic profiles at immune-relevant loci.

### Dairy breeds and population-level impact

The elevated seroprevalences in Turkish Saanen (20.89%) and Maltese (16.74%)—both dairy-purpose breeds—are consistent with European observations that intensively managed dairy goat populations accumulate higher MAP burdens [48, 63]. Several non-mutually exclusive mechanisms may contribute: higher stocking density facilitating faecal–oral transmission, more frequent introduction of replacement animals from diverse sources, nutritional stress during peak lactation potentially compromising immune function, and historical selection programmes that have prioritised production traits over disease resilience. The PAF analysis (Table 9) reframes these observations at the population level: Turkish Saanen alone accounts for 19.70% of the breed-attributable burden, followed by Maltese at 13.77%. Despite Colored Angora’s highest per-animal PR (2.702), its small population share (5.86%) limits its PAF to 9.08%. This illustrates a fundamental epidemiological principle: the breed contributing most to population-level burden is not necessarily the one with the highest individual risk but rather the one where moderate risk intersects with large population size. These findings indicate that Turkish Saanen and Maltese populations may warrant consideration as priority targets in national MAP surveillance and control programmes.

### The flat age–seroprevalence relationship

The non-significant age trend (Cochran–Armitage z = − 0.258, *p* = 0.796; Table 5) across three age categories (2–3, 4–5, ≥ 6 years) confirms that seroprevalence remains essentially stable across the productive lifespan of these animals. The numerically lower AP in the ≥ 6 group (12.37%) relative to the 4–5 group (15.26%) is interpretable as survivorship bias: clinically affected animals are preferentially culled before reaching advanced age, selectively enriching the oldest stratum with resilient, low-burden, or never-exposed individuals. The age × sex analysis corroborates the absence of any systematic interaction pattern.

### Limitations

The cross-sectional design precludes causal inference: observed breed-associated risk differentials may reflect management, environment, or herd history alongside intrinsic biological factors. The study population was enrolled from programme-affiliated herds that may not fully represent national variation. The substantial sex imbalance (~ 2.4% males [74/3,035]) limits sex-specific inference. The serological approach warrants particular attention as a limitation. ELISA for MAP exhibits stage-dependent sensitivity, with reported values as low as 15% during subclinical infection rising to approximately 87–90% in clinical cases [68, 73]. In goats specifically, serum ELISA sensitivity relative to fecal culture has ranged from 63 to 84% in asymptomatic animals to higher values at clinical stages [69, 73]. Furthermore, ELISA specificity may be reduced by cross-reactivity with shared antigens from other Mycobacterium species, including *M. tuberculosis* and other members of the *M. avium* complex [29]. These limitations mean that the true MAP infection burden in this population almost certainly exceeds the observed AP, the proportion of false-negative animals is unknown without confirmatory testing, and apparent between-breed differences in seroprevalence may partly reflect breed differences in the kinetics of antibody responses rather than differences in infection rates per se. In addition, the 31-herd cluster count may limit the stability of the cluster-robust standard error estimates in Model D, and this should be considered when interpreting confidence intervals from that model.

### Policy implications

Türkiye lacks a national paratuberculosis control programme for small ruminants—a situation starkly at odds with the 93.5% herd-level seropositivity documented here and the structured control frameworks implemented in 48 countries worldwide [48]. The present findings provide an evidence base for considering the following targeted measures. Priority actions supported by the data include: (i) the 93.5% herd-level seropositivity and near-universal endemic distribution support incorporation of MAP testing into the existing national small ruminant breeding programmes; (ii) the elevated PRs observed in Turkish Saanen and Maltese, combined with their large population shares (PAF: 19.70% and 13.77% respectively), support restriction of inter-herd animal movements without documented MAP status, particularly for these breeds; (iii) the breed-stratified SP% distributions documented in Table 4 provide a data foundation for development of breed-specific ELISA alert thresholds; and (iv) the striking seroprevalence contrast between White Angora (0%) and Colored Angora (22.47%) identifies a scientifically informative natural experiment warranting controlled exposure studies and comparative genomic investigations. The zoonotic dimension—MAP’s documented pasteurisation resistance [11] and putative role in Crohn’s disease [23, 24]—adds relevance to these recommendations within a One Health framework.

## Conclusions

This study presents the first multi-breed national serosurvey of paratuberculosis in Turkish goat populations. An apparent prevalence of 14.07% at the individual level and 93.5% at the herd level indicate endemic dissemination. Modified Poisson regression identified four breeds with significantly elevated seroprevalence: Colored Angora (PR = 2.702), Turkish Saanen (PR = 2.571), Maltese (PR = 2.027), and Honamlı (PR = 1.469), three of which retained significance under cluster-robust estimation. PAF analysis identified Turkish Saanen (19.70%) and Maltese (13.77%) as the largest population-level contributors. The complete seronegativity of white Angora alongside an apparent prevalence of 22.47% in Colored Angora represents a notable divergence whose explanation —whether reflecting differential exposure, management practices, or breed-level susceptibility— remains to be determined through controlled investigation. Interpretation of all findings is subject to the inherent limitations of cross-sectional ELISA-based serology, including stage-dependent sensitivity and potential cross-reactivity. Collectively, these findings underscore the need for national surveillance infrastructure and identify breed-level variation in MAP susceptibility as a priority area for future longitudinal and experimental research.

## Supplementary Information

Supplementary Material 1.

## Data Availability

No datasets were generated or analysed during the current study.
