# Supplementary material for: Nationwide seroprevalence of paratuberculosis in indigenous Turkish goat breeds reveals extensive herd-level circulation and breed-associated risk
Source: Ir Vet J. 2026 Jun 16;79:34. doi: 10.1186/s13620-026-00349-z (PMC13508357; doi:10.1186/s13620-026-00349-z)
Supplement: Supplementary file 1 [file 13620_2026_349_MOESM1_ESM.pdf]

Supplementary Material

First National Multi-Breed Serosurvey of Paratuberculosis in Turkish Goat Populations

Yaman et al.

Contents

Supplementary Methods
SM1. Clopper–Pearson Confidence Interval
SM2. Modified Poisson Regression: Model Specification
SM3. Sandwich (Huber–White) Robust Covariance Estimator
SM4. Intraclass Correlation Coefficient (ICC)
SM5. Cluster-Robust Variance Estimator
SM6. Cochran–Armitage Test for Trend

Supplementary Methods

SM1. Clopper–Pearson Confidence Interval

Apparent seroprevalence (AP%) was estimated as the proportion of seropositive animals within each stratum. Exact binomial 95% confidence intervals were computed using the Clopper–Pearson method [57, 58]:

$$\hat{AP} = x / n$$
$$CI\_lower = B(\alpha/2;\ x,\ n-x+1)$$
$$CI\_upper = B(1-\alpha/2;\ x+1,\ n-x)$$

where x denotes the number of seropositive animals, n the group size, $\alpha = 0.05$, and $B(\cdot)$ denotes the quantile function of the Beta distribution. This interval preserves the nominal coverage probability for small and moderate cell sizes without requiring a normal approximation, making it preferable to Wald or Wilson asymptotic alternatives in prevalence estimation with sparse strata.

SM2. Modified Poisson Regression: Model Specification

To estimate Prevalence Ratios (PRs) directly, modified Poisson regression with a log link function was applied [59, 60]. The model is specified as:

$$\log(\mu_i) = X_i\beta$$

where μ_i is the expected probability of seropositivity for animal i, and X_i is the row vector of covariates. Parameter estimates were obtained via iteratively reweighted least squares (IRLS) with convergence criterion:

$$\max|\Delta\beta| < 10^{-8} \quad \text{(maximum 200 iterations)}$$

Because Poisson variance does not correspond to binomial variance, naïve standard errors are misspecified. Accordingly, the sandwich (Huber–White) robust covariance estimator was applied in all models (see SM3).

SM3. Sandwich (Huber–White) Robust Covariance Estimator

For individual-level robust standard errors (Models A and B), the sandwich covariance matrix is:

$$V\_robust = H^{-1} \left[\Sigma_i U_i U_i^T \right] H^{-1}$$

where H = X^T diag(μ) X is the observed information matrix, and $U_i = X_i(y_i - \mu_i)$ is the individual score contribution.

SM4. Intraclass Correlation Coefficient (ICC)

The ICC for herd-level clustering was estimated using the one-way random-effects ANOVA method, quantifying the proportion of total variance attributable to between-herd differences [61]:

$$ICC = (MS\_B - MS\_W) / (MS\_B + (k_0 - 1) \cdot MS\_W)$$

where MS_B and MS^W denote between- and within-cluster mean squares, respectively, and k_0 is the design-effect–adjusted cluster size accounting for unequal herd sizes:

$$k_0 = [n\_total - (\Sigma\ n\_c^2 / n\_total)] / (C - 1)$$

where n_c is the size of cluster c and C is the number of clusters. ICC values < 0.05 were interpreted as negligible clustering, 0.05–0.20 as moderate, and > 0.20 as high.

SM5. Cluster-Robust Variance Estimator

For Model D, the sandwich covariance matrix was computed using herd as the clustering unit to account for non-independence of animals within herds:

$$V\_cluster = H^{-1} \left[\Sigma\_c\ U\_c\ U\_c^T \right] H^{-1}$$

$$U\_c = \Sigma\_\{i \in c\}\ X_i\ (y_i - \mu_i)$$

where the sum Σ_c is taken over C = 31 analytical herds. With a modest number of clusters (C = 31), cluster-robust standard errors are somewhat less stable than in large-cluster settings; this limitation is discussed in Section 4.7 of the main text.

SM6. Cochran–Armitage Test for Trend

The Cochran–Armitage test was applied to evaluate whether the proportion of seropositive animals changed monotonically across three ordinal age groups (2–3, 4–5, ≥6 years):

$$Z\_CA = \frac{\Sigma_i\ s_i\ x_i - n_i\ \bar{p} \cdot \Sigma_i\ s_i}{\sqrt{[\ \bar{p}(1-\bar{p})\ (\Sigma_i\ n_i\ s_i^2 - (\Sigma_i\ n_i\ s_i)^2 / n\_total)\ ]}}$$

where s_i are the ordered integer scores assigned to each age stratum (s = 1, 2, 3), x_i and n_i are the number of positives and total animals in stratum i, $\bar{p}$ is the overall proportion of positives, and the test statistic Z_CA follows a standard normal distribution under H_0 (equivalent to $\chi^2(1)$).
